# The *Plasmodium knowlesi* Pk41 surface protein diversity, natural selection, sub population and geographical clustering: a 6-cysteine protein family member

**DOI:** 10.7717/peerj.6141

**Published:** 2018-12-14

**Authors:** Md Atique Ahmed, Ki-Back Chu, Fu-Shi Quan

**Affiliations:** 1 Department of Medical Zoology, School of Medicine, Kyung Hee University, Seoul, Republic of Korea; 2 Department of Biomedical Science, Graduate School, Kyung Hee University, Seoul, Republic of Korea; 3 Biomedical Science Institute, Kyung Hee University, Seoul, Republic of Korea

**Keywords:** *Plasmodium knowlesi*, Pk41, Natural selection, 6-Cysteine family, s48/45 domain, Polymorphism, Malaysia, Clinical samples, Low diversity, Vaccine

## Abstract

**Introduction:**

The zoonotic malaria parasite *Plasmodium knowlesi* has currently become the most dominant form of infection in humans in Malaysia and is an emerging infectious disease in most Southeast Asian countries. The P41 is a merozoite surface protein belonging to the 6-cysteine family and is a well-characterized vaccine candidate in *P. vivax* and *P. falciparum*; however, no study has been done in the orthologous gene of *P. knowlesi*. This study investigates the level of polymorphism, haplotypes and natural selection of *pk41* genes in clinical isolates from Malaysia.

**Method:**

Thirty-five full-length *pk41* sequences from clinical isolates of Malaysia along with four laboratory lines (along with H-strain) were downloaded from public databases. For comparative analysis between species, orthologous *P41* genes from *P. falciparum, P. vivax*, *P. coatneyi* and *P. cynomolgi* were also downloaded. Genetic diversity, polymorphism, haplotype and natural selection were determined using DnaSP 5.10 software. Phylogenetic relationships between *Pk41* genes were determined using MEGA 5.0 software.

**Results:**

Analysis of 39 full-length *pk41* sequences along with the H-strain identified 36 SNPs (20 non-synonymous and 16 synonymous substitutions) resulting in 31 haplotypes. Nucleotide diversity across the full-length gene was low and was similar to its ortholog in *P. vivax*; *pv41*. Domain-wise amino acid analysis of the two s48/45 domains indicated low level of polymorphisms for both the domains, and the glutamic acid rich region had extensive size variations. In the central domain, upstream to the glutamate rich region, a unique two to six (K-E)_n_ repeat region was identified within the clinical isolates. Overall, the *pk41* genes were indicative of negative/purifying selection due to functional constraints. Domain-wise analysis of the s48/45 domains also indicated purifying selection. However, analysis of Tajima’s D across the genes identified non-synonymous SNPs in the s48/45 domain II with high positive values indicating possible epitope binding regions. All the 6-cysteine residues within the s48/45 domains were conserved within the clinical isolates indicating functional conservation of these regions. Phylogenetic analysis of full-length *pk41* genes indicated geographical clustering and identified three subpopulations of *P. knowlesi*; one originating in the laboratory lines and two originating from Sarawak, Malaysian Borneo.

**Conclusion:**

This is the first study to report on the polymorphism and natural selection of *pk41* genes from clinical isolates of Malaysia. The results reveal that there is low level of polymorphism in both s48/45 domains, indicating that this antigen could be a potential vaccine target. However, genetic and molecular immunology studies involving higher number of samples from various parts of Malaysia would be necessary to validate this antigen’s candidacy as a vaccine target for *P. knowlesi*.

## Introduction

Malaria is a major public health concern as it causes the death of a half a million people around the globe annually (WHO, 2017). The zoonotic malaria parasite *Plasmodium knowlesi* is now considered as the fifth *Plasmodium* species capable of infecting humans, and cases are rapidly emerging in many countries of Southeast Asia (Ahmed & Cox-Singh, 2015; Cox-Singh et al., 2008; Garnham, 1966). According to latest reports, *P. knowlesi* accounts for 70–80% of all malaria cases in Malaysian Borneo (Barber et al., 2017; Barber et al., 2011; Daneshvar et al., 2009; William et al., 2013) and as per the world malaria report 2017, there is a rapid increase of human cases in Malaysia (WHO, 2017). *P. knowlesi* is often misdiagnosed as *P. malariae* under the microscope, therefore molecular testing using PCR for confirmation is essential (Cox-Singh et al., 2008). The erythrocytic cycle of this parasite is 24 h, which is the fastest among all human malarias, and increase in parasite count has been associated with severe malaria in humans with fatal outcome (Cox-Singh et al., 2008; Daneshvar et al., 2009; Willmann et al., 2012). Genetic studies on invasion genes as well as whole genome studies have identified at least three subpopulations of the parasite in clinical samples from Malaysia and two of the populations were associated with the monkey hosts; *Macaca fascicularis* and *Macaca nemestrina* in Malaysian Borneo (Ahmed et al., 2014, 2016; Assefa et al., 2015; Pinheiro et al., 2015). A recent study on two genes that have been extensively used for phylogenetic studies that is the mitochondrial cytochrome oxidase I (cox 1) and smaller subunit ribosomal rRNA of *P. knowlesi* from clinical samples and wild macaques identified two distinct subpopulation which clustered geographically to Peninsular Malaysia and Malaysian Borneo (Yusof et al., 2016). These studies indicate that *P. knowlesi* infections in humans is complex and involves multiple subpopulations of the parasite and some of the infections may cause severe disease.

Antimalarial vaccine is an important tool for malaria control and elimination, but high polymorphism displayed by field isolates within candidate antigens remains as one of the major factor hindering vaccine development. Leading vaccine antigens that are found to be under positive (diversifying) selection (like ama1, msp1 and msp2) are parasite surface proteins and have evolved extensive genetic diversity in order to evade host immune response in natural parasite populations (Barry & Arnott, 2014; Patel et al., 2017; Takala et al., 2009). These candidates when tested in the field show allele-specific immune response thereby reducing the efficacy of the candidate antigen in endemic regions (Osier et al., 2010). For example, currently RTS,S, is the only candidate for vaccine development which is designed based on the *P. falciparum* circumsporozoite protein. It has reached phase IV clinical trials (Coelho et al., 2017); however, has low efficacy in the field and one of the reasons was parasite diversity and allele-specific immune response observed within field isolates of *P. falciparum* (Neafsey et al., 2015). The *pkcsp* diversity in clinical isolates of Malaysia is also high and the epitope binding regions were under the influence of positive natural selection (Fong et al., 2015). Many such antigens in *P. falciparum* (Barry et al., 2013; Genton et al., 2002; Patel et al., 2017; Soe et al., 2017) and *P. vivax* (Lo et al., 2017) have high nucleotide diversity, leading to allele-specific immune responses or low efficacy during vaccine trials. Thus, it is important to assess the level of diversity, type of natural selection and significance toward further studying of the antigen as a vaccine candidate.

Among merozoite invasion proteins widely studied in *P. falciparum* and *P. vivax*, the 6-cysteine protein family is a mutli-stage conserved surface proteins which are expressed in both asexual (merozoites) and sexual (gametocytes) stages of the parasite (Angel et al., 2008; Garcia et al., 2009). The protein family in *P. falciparum* consists of five members; Pf12, Pf12P, Pf38, Pf41 and Pf92 proteins which are expressed at the asexual stages of the parasite’s life cycle and is characterized by the presence of six conserved cysteine residues called the s48/45 domains (Arredondo & Kappe, 2017; Garcia et al., 2009). The protein has a signal peptide but lacks the transmembrane domain (Garcia et al., 2009). The Pf41 has two high affinity binding peptides in the s48/45 domains and is anchored to the merozoite surface by forming an inverted heteroduplex with Pf12 (Taechalertpaisarn et al., 2012; Tonkin et al., 2013). Recent structural studies on Pf41 has revealed that there is an intra-domain insertion which is necessary for binding to Pf12 and this protects it from proteolytic cleavage (Parker, Peng & Boulanger, 2015). The *P. vivax* ortholog *pv41* gene has been characterized (Angel et al., 2008). The Pv41 is localized at the merozoite surface and high immunogenicity observed from patient sera suggests its exposure to the host’s immune system (Cheng et al., 2013). Pf41 has been under positive natural selection (Tonkin et al., 2013) and recognized by serum from naturally infected individuals with seroprevalences in the range of 32–88% (Osier et al., 2014; Richards et al., 2013). Population genetic analysis has revealed positive balancing selection for C-terminal regions of the *pv41* genes from China–Myanmar area indicating immune evasion by the parasite (Wang et al., 2014). Despite the significance of these studies, which has demonstrated the potential of P41 as a vaccine candidate, no study has been conducted in the *P. knowlesi* ortholog Pk41 protein.

In this study, the domains of Pk41 protein were characterized based on the amino acid sequence alignment to its orthologs in *P. vivax* (Pv41) and *P. falciparum* (Pf41). Genetic diversity, natural selection, number of haplotypes and haplotype diversity within 39 isolates [35 clinical isolates and four laboratory lines (along with the H-strain)] from Malaysia were determined using full-length *Pk41* genes. Since this is the first study of Pk41 from clinical samples, the information obtained from this study will be helpful to understand the level of polymorphism within the functional domains in field isolates for future functional studies as well as rational design and formulation of a blood-stage vaccine against *P. knowlesi*.

## Materials and Methods

### Pk41 sequence data

Thirty-nine full-length *pk41* gene sequences were obtained from published database from clinical isolates originating from Malaysian Borneo and three long-time isolated lines from Peninsular Malaysia (along with the H-strain, PKNH_0303000) and the Philippine Strain (Table S1) (Assefa et al., 2015). The genomes were downloaded from the European Nucleotide Archive (https://www.ebi.ac.uk/ena). A map showing the geographical location of the isolates used in the study is shown in Fig. S1. Signal peptide for the full-length Pk41 was predicted using Signal IP 3.0 prediction software (Petersen et al., 2011). Sequence data were aligned using the CLUSTAL-W program in MegAlign Lasergene v 7.0 (DNASTAR) and polymorphism and phylogenetic analyses were conducted in MEGA 5.0 software. In order to determine the relationship between Pk41 sequences (laboratory lines and clinical isolates from Sarawak, Malaysian Borneo), phylogenetic analyses were conducted using deduced amino acid sequences using Maximum Likelihood (ML) method based on Poisson correction model as described in MEGA 5.0 with 1,000 bootstrap replicates to test the robustness of the trees (Table S1). Orthologous members of *P. falciparum* (PF3D7_04049000), *P. cynomolgi* (PCYB_031270), *P. coatneyi* (PCOAH_00006000) and *P. vivax* Sal-1 (PVX_000995) were also included in the analyses. ML-based phylogenetic trees were also constructed based on the s48/41 domain I and II of Pk41 protein.

### Sequence diversity and natural selection

Sequence diversity (π), which is defined as the average number of nucleotide differences per site between two sequences was determined by DnaSP v5.10 software (Librado & Rozas, 2009). Number of polymorphic sites, parsimony informative sites (sites that have a minimum of two nucleotides that are present at least twice), number of synonymous (silent mutations) and non-synonymous substitutions (replacement mutations or mutations leading to change in amino acids), number of haplotypes (H), singletons (a nucleotide variant that appears only once in among the sequences) and haplotype diversity within the *pk41* sequences were also determined by DnaSP software. Graphical representation of nucleotide diversity was conducted using the same software with window length 50 bp and step size 12 bp.

Natural selection was determined by calculating the rates of synonymous substitutions per synonymous site (dS) and nonsynonymous substitutions per nonsynonymous site (dN) which were computed by using Nei & Gojobori’s method (1986). Juke-Cantor correction and their standard errors of these parameters were estimated by the bootstrap method with 1,000 pseudo replicates as implemented in the MEGA 5.0 program (Tamura et al., 2011). Additionally, the Tajima’s D, Fu & Li’s D* and F* neutrality tests were performed as implemented in DnaSP v5.10 software. Tajima’s D is expected to be zero under neutrality. When Tajima’s D values are positive and significant it indicate positive/balancing selection, whereas negative values suggest negative selection or population expansion. Graphical representation of Tajima’s D was also conducted using the same software. Significant positive values for Fu & Li’s D* and F* also indicates population contraction due to a selection event while negative values indicated population expansion and excess of singletons.

### Genetic differentiation

The ARLEQUIN software v.3.5.1.3 (Excoffier, Laval & Schneider, 2007) was used to compute pairwise differences (*F_ST_*) between *P. knowlesi* subpopulations which were identified though ML-based phylogenetic analysis. The *F_ST_ values were determined* with 10,100 permutations. *F_ST_* is a comparison of the sum of genetic variability within and between populations based on the differences in allelic frequencies. *F_ST_* values are interpreted as no (0), low (>0–0.05), moderate (0.05–0.15) and high (0.15–0.25) genetic differentiation.

## Results

### Pk41 sequence identity within ortholg members and diversity within *P. knowlesi* population

**Figure 1:**
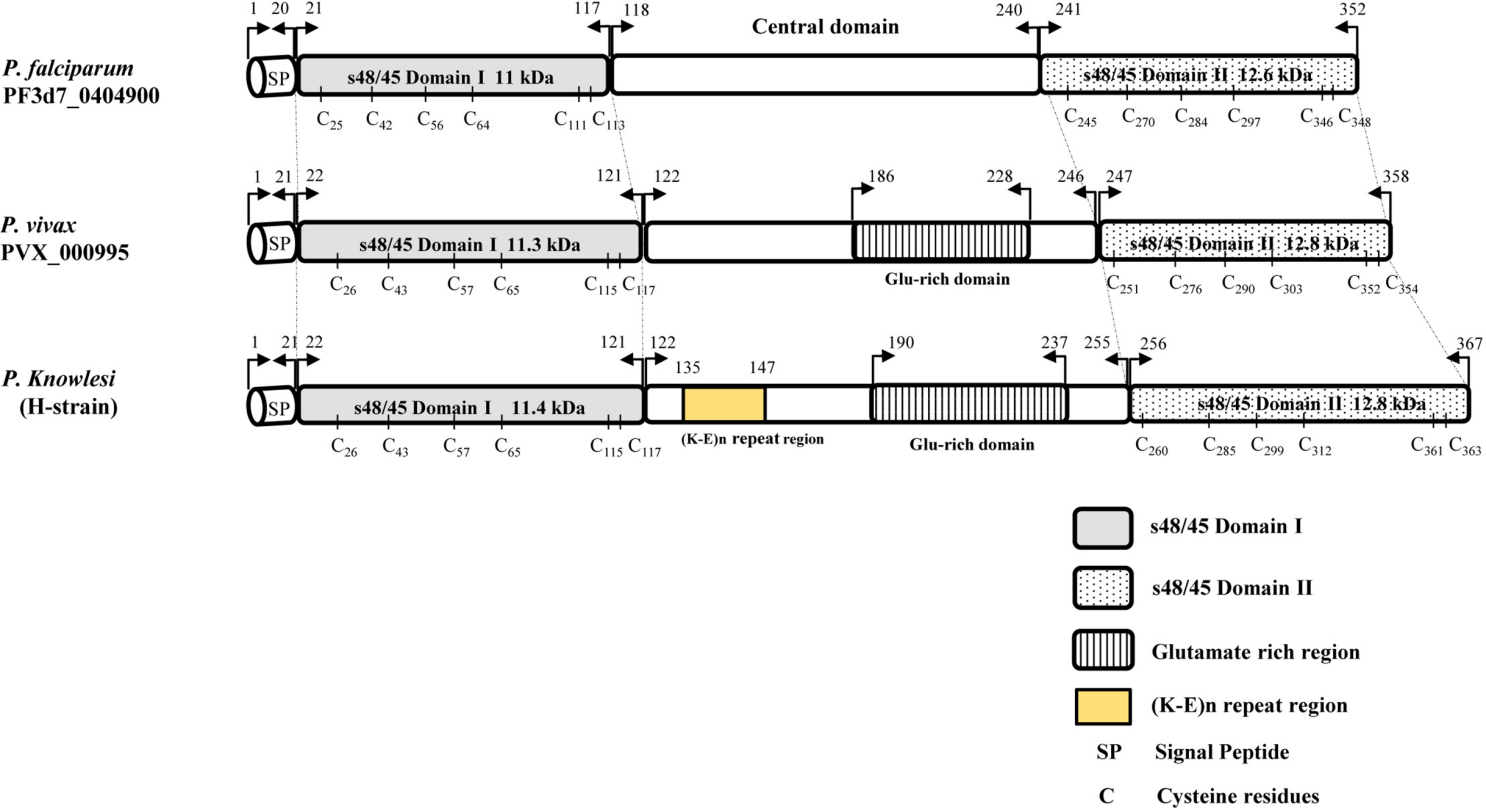
Schematic structure of P41 proteins in *P. falciparum, P. vivax* and *P. knowlesi*. The conserved two 6-Cys domains, labeled as s48/45 Domain I and II along with its molecular weight, are in shaded and dotted background, respectively. (K-E)n repeat region is shown in yellow. The position of cystiene residues are given. The arrow marks indicate amino acid co-ordinates for each domain.

**Figure 2:**
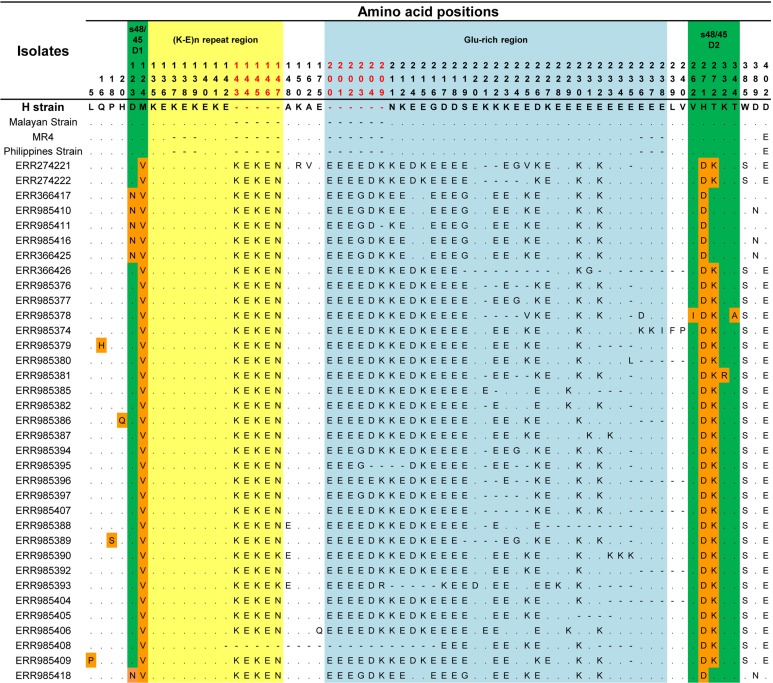
Amino acid polymorphism within Pk41 proteins sequences from Malaysia. The green shaded regions represents the polymorphism with s48/45 domains I and II and the polymorphic residues are shaded in orange. The yellow shaded region is the (K-E) repeat region and blue shaded region is the Glu-rich region and the polymorphism within them. The amino acid position colored in red represents the insertions within the clinical isolates from Malaysian Borneo.

The signal peptide of the Pk41 protein was detected between amino acid positions 21 and 22 using the Signal IP server (Fig. S2). There was no transmembrane domain predicted within the alignment. Alignment and comparison of the amino acid sequences of the full-length *P. knowlesi* H reference strain Pk41 sequences with its ortholog in *P. vivax* Sal-1 reference and *P. falciparum* 3D7 reference strain showed 84.9% and 41.5% identities respectively. The sequence identity differed mainly due to the central region which had a glutamate rich region in *P. vivax* and *P. knowlesi* but absent in *P. falciparum*. The schematic structure of *pk41* gene with domain coordinates and 6-cysteine residues in comparison with its orthologs in *P. vivax* and *P. falciparum* are shown in Fig. 1. Other orthologs in primate malarias that is *P. cynomolgi* B strain and *P. coatneyi* Hackeri strain showed 84.1% and 87.5% sequence identity respectively. Within the full-length *pk41* sequences (*n* = 39), there were 36 polymorphic sites which led to 20 synonymous and 16 non-synonymous substitutions. There were 28 parsimony informative sites, of which three sites were of three variants and 18 singleton variable sites leading to 31 haplotypes (Table S2). All the 6-cysteine residues within the two s48/45 domains were conserved, indicating active functional binding to host erythrocytes (Figs. S3 and S4). In addition to non-synonymous SNPs, the Pk41 had a repeat region encoding Lysine-Glutamic acid (K-E)_n_ region (Fig. 2). The number of repeats varied from two to six within the laboratory lines and Sarawak, Malaysian Borneo. In addition to the (K-E)_n_ repeat units, variation of glutamic acid (E)_n_ repeats within the central domain led to size variations of 1,182–1,233 bp within the isolates (Fig. 2).

**Figure 3:**
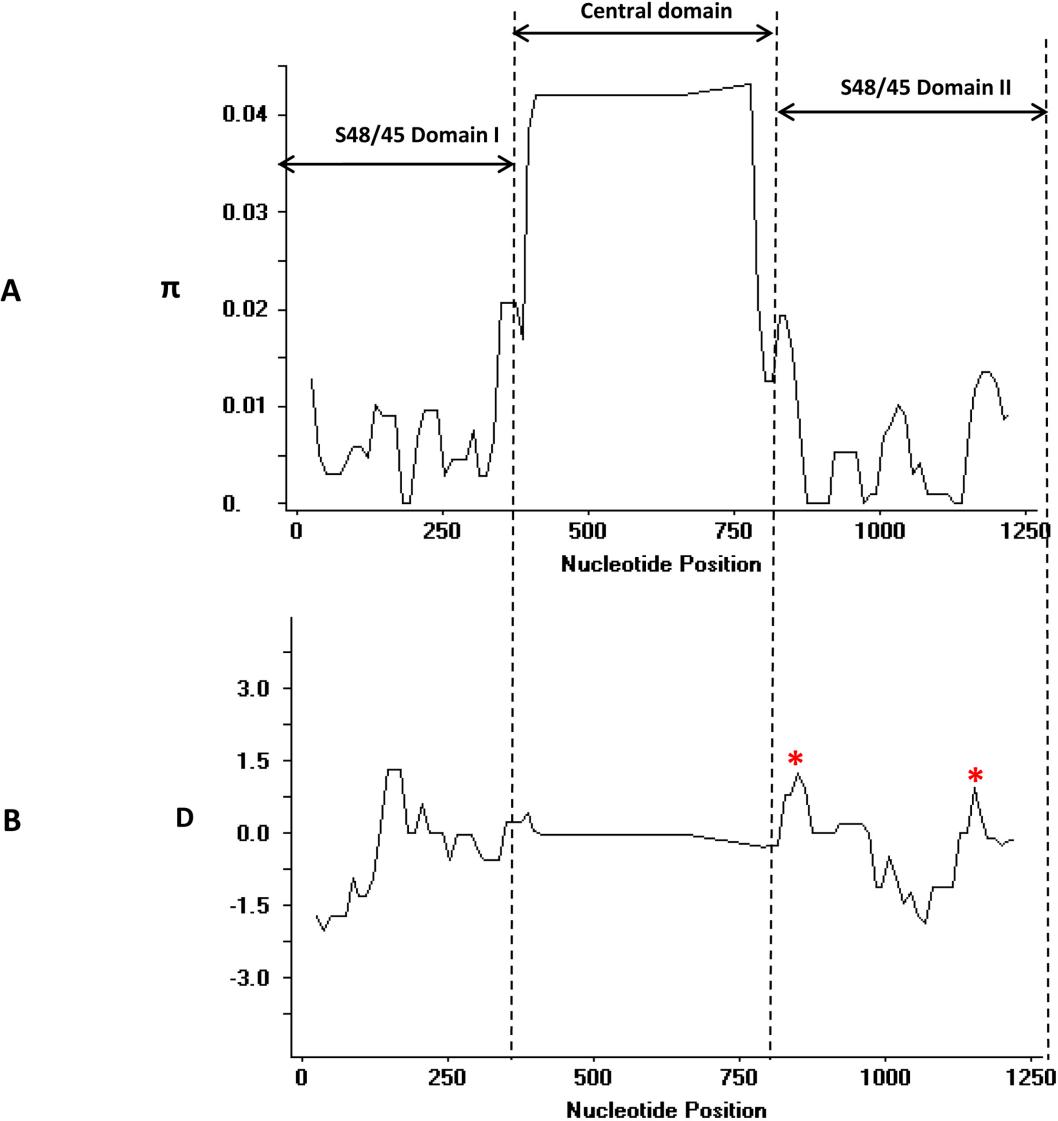
(A) Graphical representation of nucleotide diversity (π) within Pk41 genes and (B) Tajimas D value across the full-length Pk41 genes. The three domains; s48/45 domain I, II and the central domains are marked and with double-sided arrows. Dotted lines were used to indicate the peaks in π graph and the D graph. The asterisk indicates high positive Tajimas D values within the domain. The π graph and the D graph were drawn with window length 50 and step size 12 in Dnasp.

The overall nucleotide diversity of full-length *pk41* was higher (π = 0.00959 ± SD 0.0001) compared to its ortholog in *P. vivax* (Forero-Rodriguez, Garzon-Ospina & Patarroyo, 2014) (Table 1). However, high nucleotide diversity within *pk41* could be attributed to the size variations observed within the (K-E)_n_ repeat region and the Glu-rich region within the *P. knowlesi* clinical isolates. The graphical representation of nucleotide diversity using the sliding window plot (window length 50 bp and step size 12 bp) also revealed that the diversity was high only within the central domain region due to the size variations (Fig. 3A). The total number of insertion–deletion (InDel) sites within the central domain was found to be 172. Of the 172 sites, 55 sites could be analyzed and the indel diversity was found to be high [π (*i*) = 0.354]. Most SNPs identified within these s48/45 domains were synonymous (Table 1). Domain-wise analysis of the s48/45 domain I and domain II indicated that both domains had very low levels of polymorphisms similar to its ortholog in *P. vivax* (Forero-Rodriguez, Garzon-Ospina & Patarroyo, 2014). Only two non-synonymous mutations N123D and M124V were identified within the s48/45 domain I (Fig. 2), which had minor allele frequency (MAF) greater than 10%. The s48/45 domain II had five mutations, of which only two non-synonymous mutations with MAF > 10% (H271D and T272K) were identified (Fig. 2). The remaining three mutations V262I, K332R and T344A were singleton non-synonymous mutations (Fig. 2). The two s48/45 domains shared similar levels of nucleotide diversity and it ranged from π = 0.00582–0.00530. The domain I had a slightly higher haplotype diversity compared to domain II (Table 1).

**Table 1:** Estimates of nucleotide diversity, haplotype diversity and neutrality indices of *pk41*.

Domain	No. samples	SNPs	Syn	NonSyn	No. haplotype	Diversity ± SD		Taj D	dN-dS ± S.E	Fu & Li’s D*	Fu & Li’s F*
						Haplotype	Nucleotide				
Full-length	39	36	20	16	31	0.982 ± 0.011	0.00959 ± 0.0001	−0.79 (*P* > 0.1)	−0.02 ± 0.006	−1.29 (*P* > 0.1)	−1.37 (*P* > 0.1)
S48/45 Domain I		12	10	2	14	0.870 ± 0.038	0.00582 ± 0.0009	−0.21 (*P* > 0.1)	−0.03 ± 0.001	0.46 (*P* > 0.1)	0.28 (*P* > 0.1)
Glutamic-rich region (excluding InDel sites)		9	7	2	10	0.750 ± 0.060	0.00543 ± 0.0007	−1.31 (*P* > 0.1)	0.002 ± 0.0001	−2.06 (*P* > 0.1)	−2.14 (*P* > 0.1)
S48/45 Domain II		10	5	5	9	0.727 ± 0.064	0.00530 ± 0.0007	−0.71 (*P* > 0.1)	−0.013 ± 0.008	−1.48 (*P* > 0.1)	−1.45 (*P* > 0.1)

**Note:**

SNPs, Single nucleotide polymorphisms; SD, Standard deviation: Syn, Synonymous substitutions; NonSyn, Non synonymous substitutions: SE, Standard error.

### Natural selection of Pk41

Analysis for natural selection of *pk41* genes from Malaysia indicated that the gene is under purifying/negative selection, implying functional constraints. The overall dN-dS, Taj D, Fu and Li’s D* and F* values across the genes were negative (Table 1) but not significant. Domain-wise analysis of dN-dS also indicated that the s48/45 domains were under negative selection. Taj D values and Fu & Li’s D* and F* were also negative, but not significant indicating purifying selection and population expansion in each domain (Table 1). However, graphical representation of Taj D across the entire gene identified certain regions within the s48/45 domains, which showed high positive D values (Fig. 3B) indicating non-synonymous SNPs in the region could be under positive balancing selection. These results indicated that a higher number of samples will be necessary for a statistically significant negative selection at this locus.

### Phylogenetic analysis

**Figure 4:**
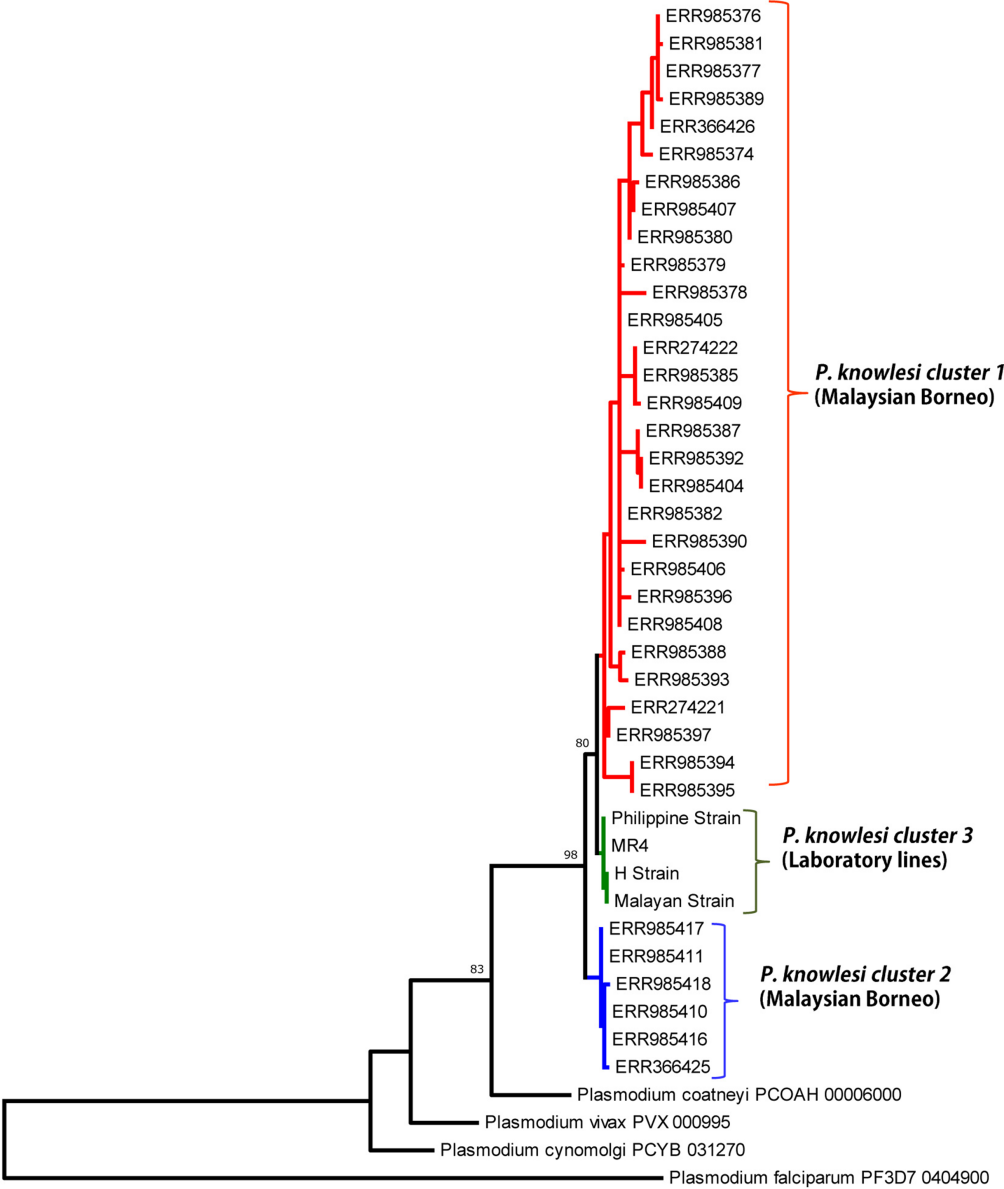
Phylogenetic relationship of Pk41 proteins from clinical isolates of Malaysia and the ortholog in other *Plasmodium* species based on Maximum Likelihood method. The two *P. knowlesi* Pk41 subpopulations identified in Malaysian Borneo are shown as cluster 1 and cluster 2 and the four laboratory lines formed the cluster 3 from Peninsular Malaysia. Numbers at the nodes indicate bootstrap values.

Phylogenetic analysis of the 39 full-length Pk41 deduced amino acid sequences with other *Plasmodium* species using ML method identified three distinct *P. knowlesi* sub-populations. Of the three subpopulations, two populations originated from Malaysian Borneo (Fig. 4). The four laboratory lines, the H-strain, the Malayan Strain, MR4 and the Philippine Strain, which originated from Peninsular Malaysia and Philippines formed the third subpopulation (Fig. 4). The *pk41* sequences were obtained from a previously published genomic study (Assefa et al., 2015) where the clinical samples were found to be associated with the primary hosts of *P. knowlesi*, that is *Macaca fascicularis* and *Macaca nemestrina*. Thus, cluster 1 and cluster 2 identified in this study also associated with *Macaca fascicularis* and *Macaca nemestrina*, respectively. The ML method showed that the Pk41 was more closely related to *P. coatneyi* 41 compared to its ortholog in *P. vivax*, *P. falciparum* and *P. cynomolgi*. Independent domain-wise ML-based phylogenetic analysis (for s48/45 domain I and II) also indicated same pattern of three subpopulations (Figs. S5 and S6). Two distinct amino acid polymorphisms N123D and T272K in domain I and II of s48/45, respectively, were the core determinants in separating *P. knowlesi* clinical isolates into two subpopulations in Sarawak (Fig. 2). The minor alleles (N and T) were associated with *Macaca nemestrina* and the major allele (D and K) were associated with *Macaca fascicularis*. However, phylogenetic trees constructed based on the central domain did not indicate any pattern of subpopulations (data not shown).

### Genetic differentiation within *P. knowlesi* subpopulations

Pairwise population differentiation index (*F_ST_* values) using ARLEQUIN software identified very high and significant genetic differentiation within the subpopulations originating from Sarawak, Malaysian Borneo (*F_ST_* = 0.732, *P <* 0.000, between cluster 1 and cluster 2), Table S3. Similar high and significant genetic differentiation was also observed between parasites population of within the laboratory lines and Sarawak, Malaysian Borneo clusters (Table S3) suggesting that parasitic transmission is confined to each of the regions. Previous studies have established that these two distinct sympatric subpopulation clusters from Sarawak, Malaysian Borneo were associated with their primary hosts that is *Macaca fascicularis* and *Macaca nemestrina* (Assefa et al., 2015; Divis et al., 2017).

## Discussion

Blood stage antigens, which are localized at the merozoite surface, play an important role in invasion into erythrocytes. These antigens are directly exposed to host immune response during merozoite egress, and thus are excellent vaccine candidates. A candidate antigen should optimally possess low polymorphism to be efficacious across different geographical locations and avoid allele specific immune response. Recent *P. knowlesi* studies on known vaccine candidates for example normocyte binding protein xa and xb (Ahmed et al., 2014), *csp* (Fong et al., 2015), *msp-142* (Yap et al., 2017) showed high genetic diversity in field isolates of Malaysia. However, some merozoite surface proteins for example *pkaarp* (Muh et al., 2018) and *pkmsp1p* (Ahmed, Fauzi & Han, 2018a) showed low levels of polymorphisms, which signifies potential candidacy for vaccine studies. The 6-cysteine protein family is conserved across *Plasmodium* species and Pf41 and Pv41 are major blood stage antigens that generate protective immune response in patients (Crosnier et al., 2013; Hostetler et al., 2015). Low levels of polymorphisms in field isolates have been reported and may prove to be excellent vaccine candidates (Forero-Rodriguez, Garzon-Ospina & Patarroyo, 2014). The objectives of the present study were to genetically characterize the *pk41* gene from clinical isolates from Sarawak, Malaysian Borneo and study the level of genetic diversity, natural selection acting on the full-length Pk41 and at its functional s48/45 domains. Sequence alignment of 39 full-length amino acid sequences of Pk41 showed that it shares approximately 84.9% sequence identity with its ortholog Pv41. Since *P. knowlesi* is a primate malaria parasite and closely related to *P. coatneyi* (Muehlenbein et al., 2015), its sequence identity was highest with Pco41. The overall nucleotide diversity of *pk41* was low within the clinical isolates (36 SNPs) and were of similar levels to its ortholog species *P. vivax* (Forero-Rodriguez, Garzon-Ospina & Patarroyo, 2014). Despite the fact that phylogenetic analysis of Pk41 amino acid sequences revealed three distinct subpopulations in Malaysia, the non-synonymous SNPs in each of the s48/45 domains were very low indicating functional conservation. It is interesting to note that the diversity observed within the *pk41* genes was due to the central domain which had two repeat regions that is (K-E)_n_ repeat region and the glutamic acid (E)_n_ repeat region. Insertions of two to three (K-E)_n_ repeats were detected only in isolates originating from Sarawak, Malaysian Borneo, but not in the laboratory lines which originated from Peninsular Malaysia (H-strain) and Philippines. Variation of repeat numbers that is in (K-E)_n_ and (E)_n_ led to size variations within the clinical isolates. It is also important to note that this (K-E)_n_ repeat region is unique only to *P. knowlesi* and absent in its ortholog *P. vivax* and the clinical isolate originating from Sarawak had higher number of repeats. The implications of these repeats only in clinical isolates could be due to recent evolution of *P. knowlesi* in human population and these repeat units could be potential targets for immune evasion. Thus, immunological studies considering these repeat units would be necessary to further characterize as a vaccine candidate.

Tests of natural selection (using dN-dS, Taj’s D and Li and Fu’s D* and F*) yielded negative values indicating that the Pk41 is under negative/purifying selection; however, the *P*-values were not significant indicating higher sample size would be necessary for a statistically significant result. Domain-wise analysis of natural selection also indicated negative selection. It is interesting to note that *Pv41* and *Pf41* have been found to be under positive natural selection in various geographical locations (Tonkin et al., 2013; Wang et al., 2014). The contrasting results observed in *P. knowlesi* could be because of the three subpopulations identified in this study and majority of the clinical isolates were from Sarawak where two subpopulations coexists. In addition to this, there were natural variations of repeat units (within the central domain) in the clinical isolates and not all codons could be analyzed for natural selection. Thus, it would be important to collect higher number of samples to determine natural selection within each subpopulations. In the current study, it is also worth mentioning the presence of non-synonymous SNPs within the s48/45 domain II of *pk41* showing high Taj D values, which may indicate possible epitope binding regions under immune selection pressure. However, these would need further confirmation through immunological studies targeted at these regions. Reports of positive balancing selection within the C terminal s48/45 domain II of *pv41* genes have been recently reported from an endemic region (Wang et al., 2014). Similar positive peaks for Taj D values for CTL epitope regions in *P. falciparum* and *P. knowlesi* TRAP protein have been identified (Ahmed, Lau & Quan, 2018b; Weedall et al., 2007).

The ML-based phylogenetic tree showed separation of the Pk41 proteins from Malaysian Borneo into two populations while the four laboratory lines (H-strain, Malayan strain, Philippine Strain and the MR4 strain) formed the third subpopulation originating from Peninsular Malaysia and Philippines as observed by Assefa et al. (2015). Interestingly, only s48/45 domain I and II contributed to the grouping of the parasites into three subpopulations indicating selective forces may play a decisive role in bifurcation of trees. Previous studies on blood vaccine candidates such as the PkNBPXa (Ahmed et al., 2016), PkAMA1 (Faber et al., 2015) and PkMSP1P (Ahmed, Fauzi & Han, 2018a) from Malaysian Borneo also documented geographical separation and the presence of two *P. knowlesi* subpopulations for these antigens.

Population differentiation index *F_ST_* based on Pk41 between the parasite sub-populations had significantly higher genetic differentiation (*F_ST_* > 0.7, Table S3) and this can be attributed to the sympatric nature of the parasite populations and their host associated factors as identified previously in a genomic study (Assefa et al., 2015). High genetic differentiation values between long-term isolated laboratory lines and Sarawak, Malaysian Borneo isolates were due to the geographical distance between the two regions, which is separated by the South China Sea (Fig. S1). These results indicate localized transmission in South Asian countries and Malaysian Borneo and the co-existence of the two sympatric subpopulations in Sarawak, Malaysian Borneo. However, a vaccine designed based on the low polymorphic s48/45 domains could still be effective for all the three subpopulations. Thus, further characterization through genetic as well as immunological studies is necessary.

## Conclusions

The present study is the first to investigate genetic diversity and natural selection of the *pk41* gene from clinical samples of Sarawak, Malaysia. Low level of genetic diversity was observed within the s48/45 domains of the gene, accompanied by extensive size variations due to the repeat regions in the central domain. Overall, the gene is probably under negative/purifying natural selection; however, certain regions in the s48/45 domain II showed high Tajima’s D values thus could be under balancing selection. Further genetic studies with higher number of clinical isolates (specifically form Peninsular Malaysia) as well as immunological studies characterizing the functional domains would be necessary to validate Pk41 as a potential vaccine candidate.

## Supplemental Information

10.7717/peerj.6141/supp-1 Supplemental Information 1 Map of Malaysia with geographical location of sample collection sites. Geographical location of sequences used in the study. Note: the Philippine Strain (SRR2225573) used in the study originated from the Philippines.

10.7717/peerj.6141/supp-2 Supplemental Information 2 Signal peptide prediction. Signal peptide was predicted with cleavage site between pos. 21 and 22.

10.7717/peerj.6141/supp-3 Supplemental Information 3 Conserved 6-cysteine residues within s48/45 domain I of Pk41 clinical isolates. The conserved cysteine residues are highlighted in yellow.

10.7717/peerj.6141/supp-4 Supplemental Information 4 Conserved 6-cysteine residues within s48/45 domain II of Pk41 clinical isolates. The conserved cysteine residues are highlighted in yellow.

10.7717/peerj.6141/supp-5 Supplemental Information 5 Phylogenetic relationship of Pk41 proteins (s48/45 domain I) from clinical isolates of Malaysia and the ortholog in other *Plasmodium* species based on Maximum Likelihood method. The two *P. knowlesi* sub-populations identified based on s48/45 domain I in Malaysian Borneo are shown as cluster 1 and cluster 2 and the four laboratory lines formed the cluster 3 from Peninsular Malaysia. Numbers at the nodes indicate bootstrap values.

10.7717/peerj.6141/supp-6 Supplemental Information 6 Phylogenetic relationship of Pk41 proteins (s48/45 domain II) from clinical isolates of Malaysia and the ortholog in other *Plasmodium* species based on Maximum Likelihood method. The two *P. knowlesi* sub-populations identified based on s48/45 domain II in Malaysian Borneo are shown as cluster 1 and cluster 2 and the four laboratory lines formed the cluster 3 from Peninsular Malaysia. Numbers at the nodes indicate bootstrap values.

10.7717/peerj.6141/supp-7 Supplemental Information 7 Accession number of Pk41 sequences used in the study and their geographical origin. P: Peninsular.

10.7717/peerj.6141/supp-8 Supplemental Information 8 The 31 pk41 nucleotide haplotypes identified with *P. knowlesi* clinical isolates.

10.7717/peerj.6141/supp-9 Supplemental Information 9 Population differentiation values (*F_ST_*) from each sub-populations of Malaysia based on *Pk41* genes.

## Additional Information and Declarations

### Data Availability

The following information was supplied regarding data availability: https://www.ebi.ac.uk, ERR274221, ERR274222, ERR366425, ERR366426, ERR985374, ERR985376, ERR985377, ERR985378, ERR985379, ERR985380, ERR985381, ERR985382, ERR985385, ERR985386, ERR985387, ERR985388, ERR985390, ERR985392, ERR985393, ERR985394, ERR985395, ERR985396, ERR985397, ERR985404, ERR985405, ERR985406, ERR985407, ERR985408, ERR985409, ERR985410, ERR985411, ERR985416, ERR985417, ERR985418, SRR2225571 (MR4), SRR2225573 (Philippine Strain), PKNOH_S02295300 (Malayan Strain Pk1A), PKNH_0303000 (H-strain), ERR985389.
