# Supplementary figures and images for: The Plasmodium knowlesi Pk41 surface protein diversity, natural selection, sub population and geographical clustering: a 6-cysteine protein family member
Source: PeerJ. 2018 Dec 14;6:e6141. doi: 10.7717/peerj.6141 (PMC6296336; doi:10.7717/peerj.6141)

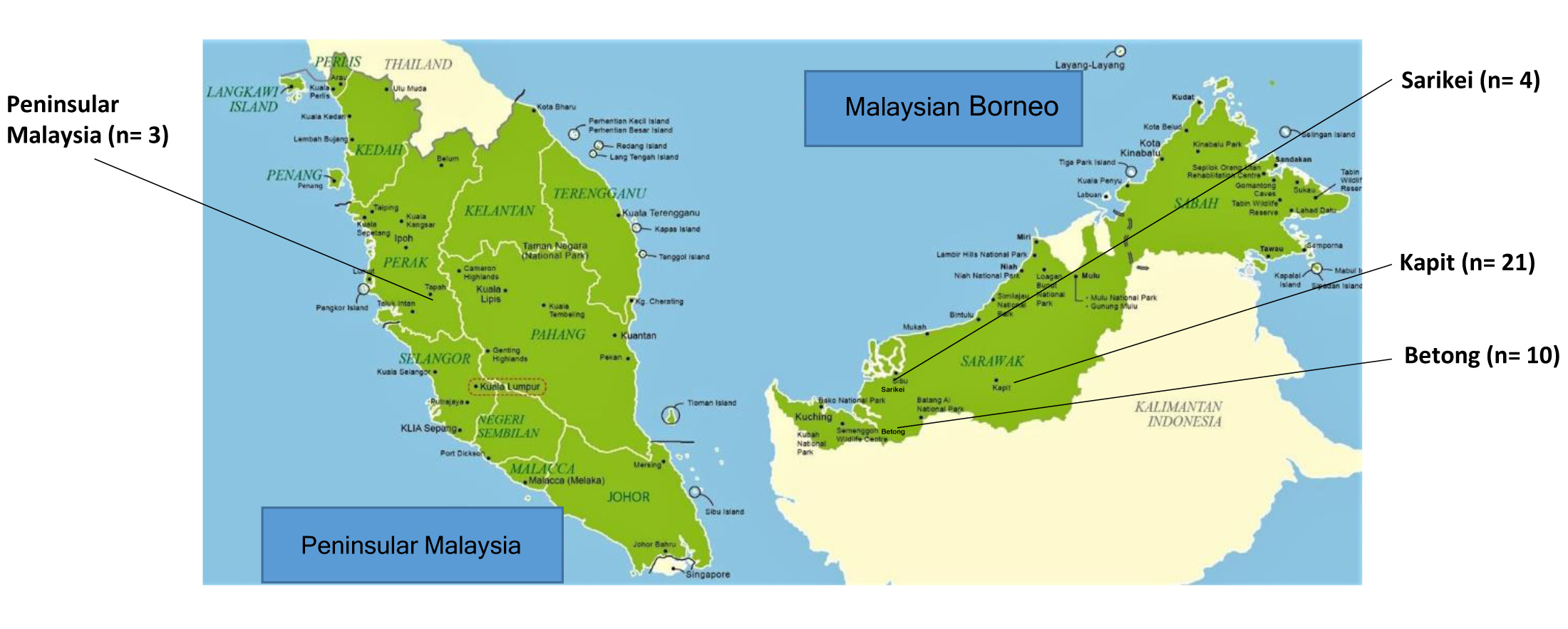

Supplement: Supplemental Information 1 — Geographical location of sequences used in the study. Note: the Philippine Strain (SRR2225573) used in the study originated from the Philippines. [file peerj-06-6141-s001.png]

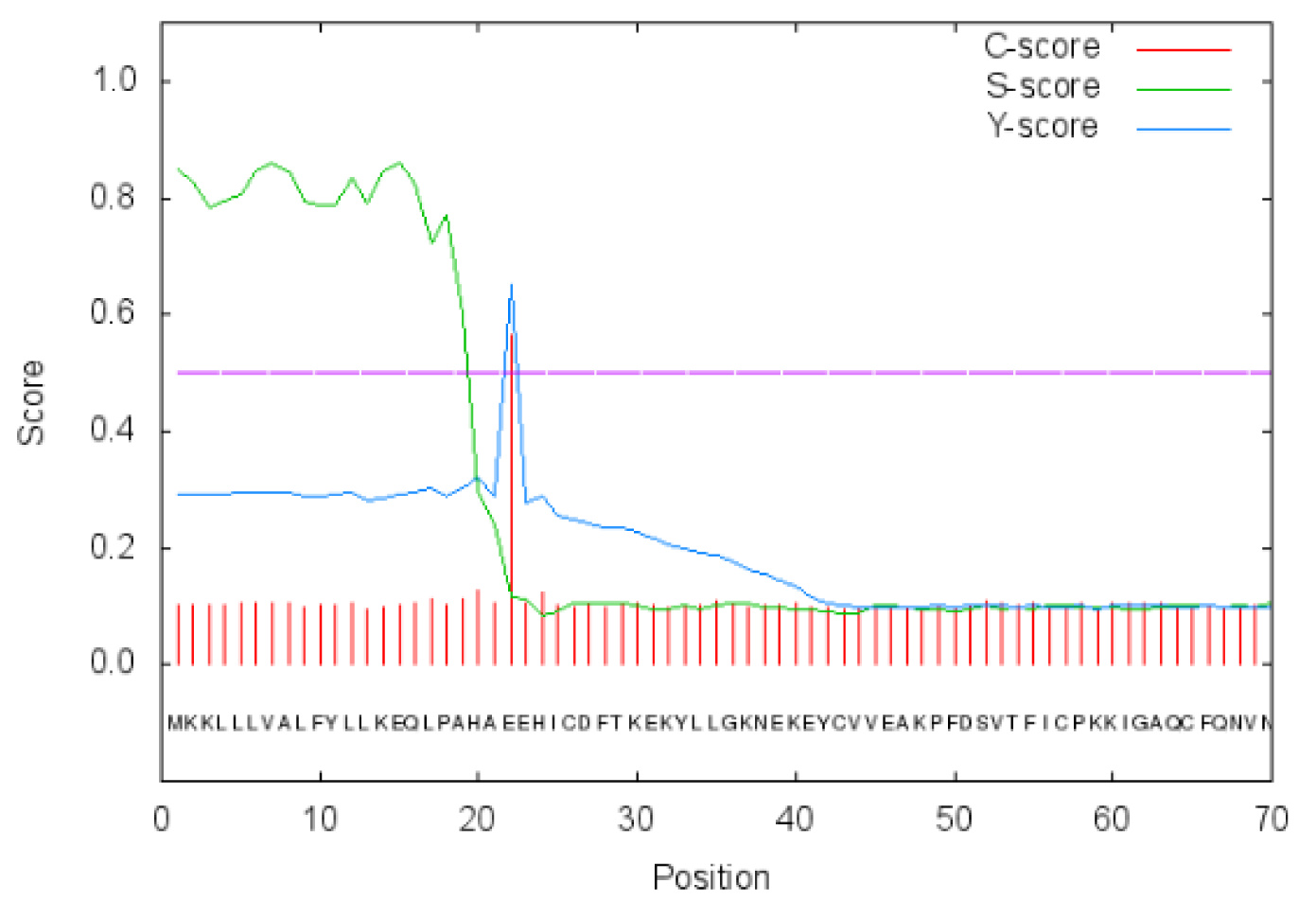

Supplement: Supplemental Information 2 — Signal peptide was predicted with cleavage site between pos. 21 and 22. [file peerj-06-6141-s002.jpg]

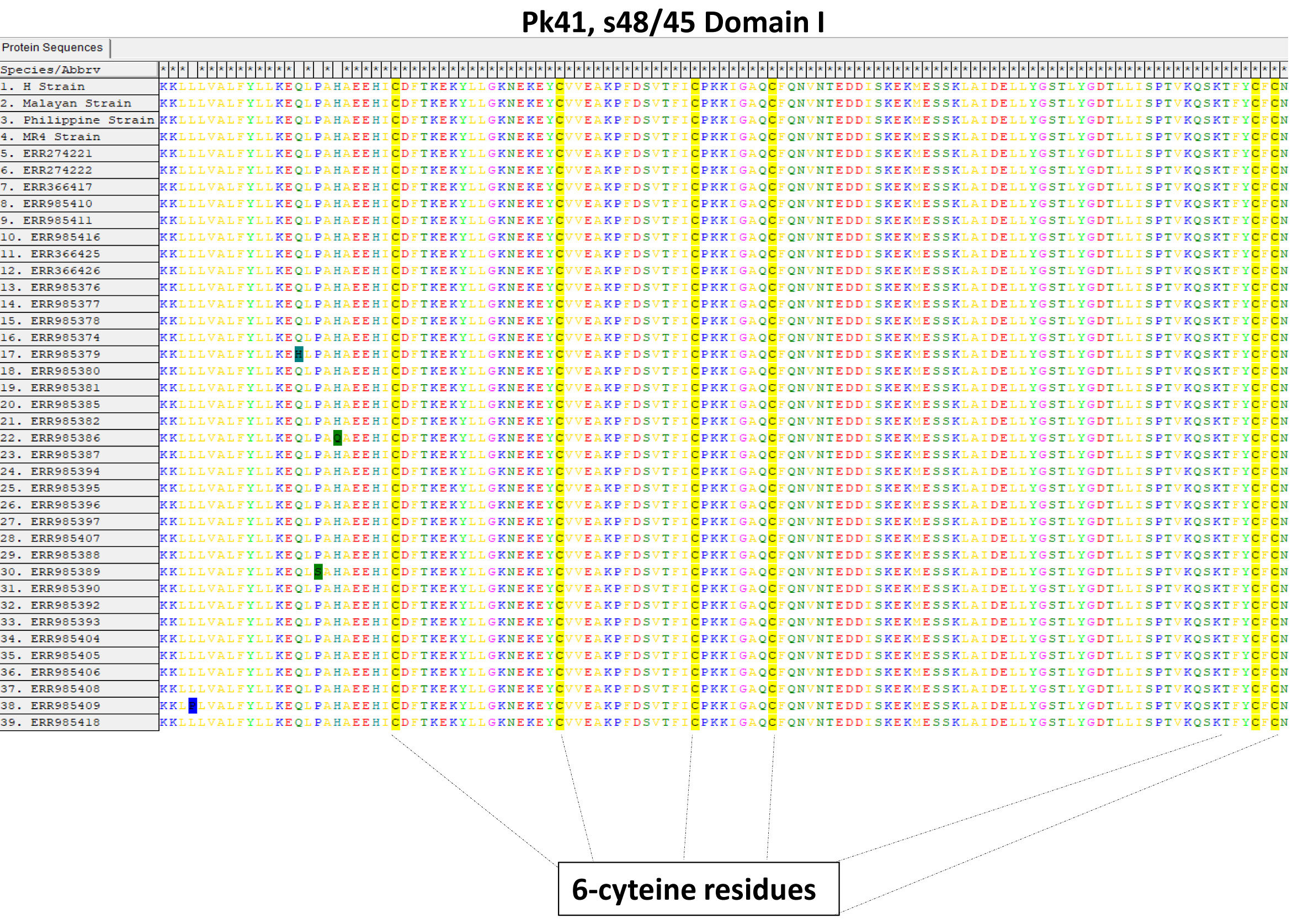

Supplement: Supplemental Information 3 — The conserved cysteine residues are highlighted in yellow. [file peerj-06-6141-s003.png]

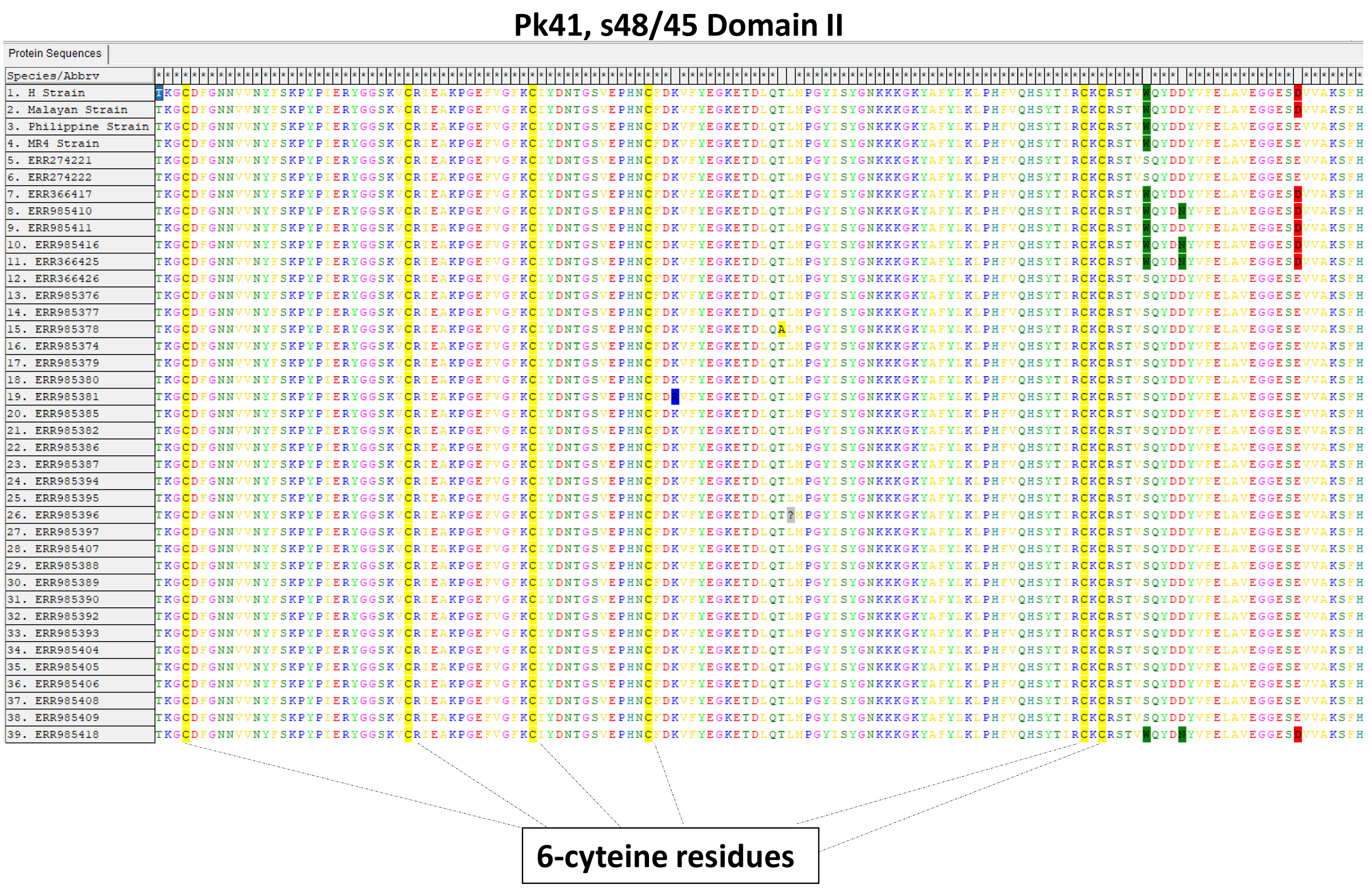

Supplement: Supplemental Information 4 — The conserved cysteine residues are highlighted in yellow. [file peerj-06-6141-s004.png]

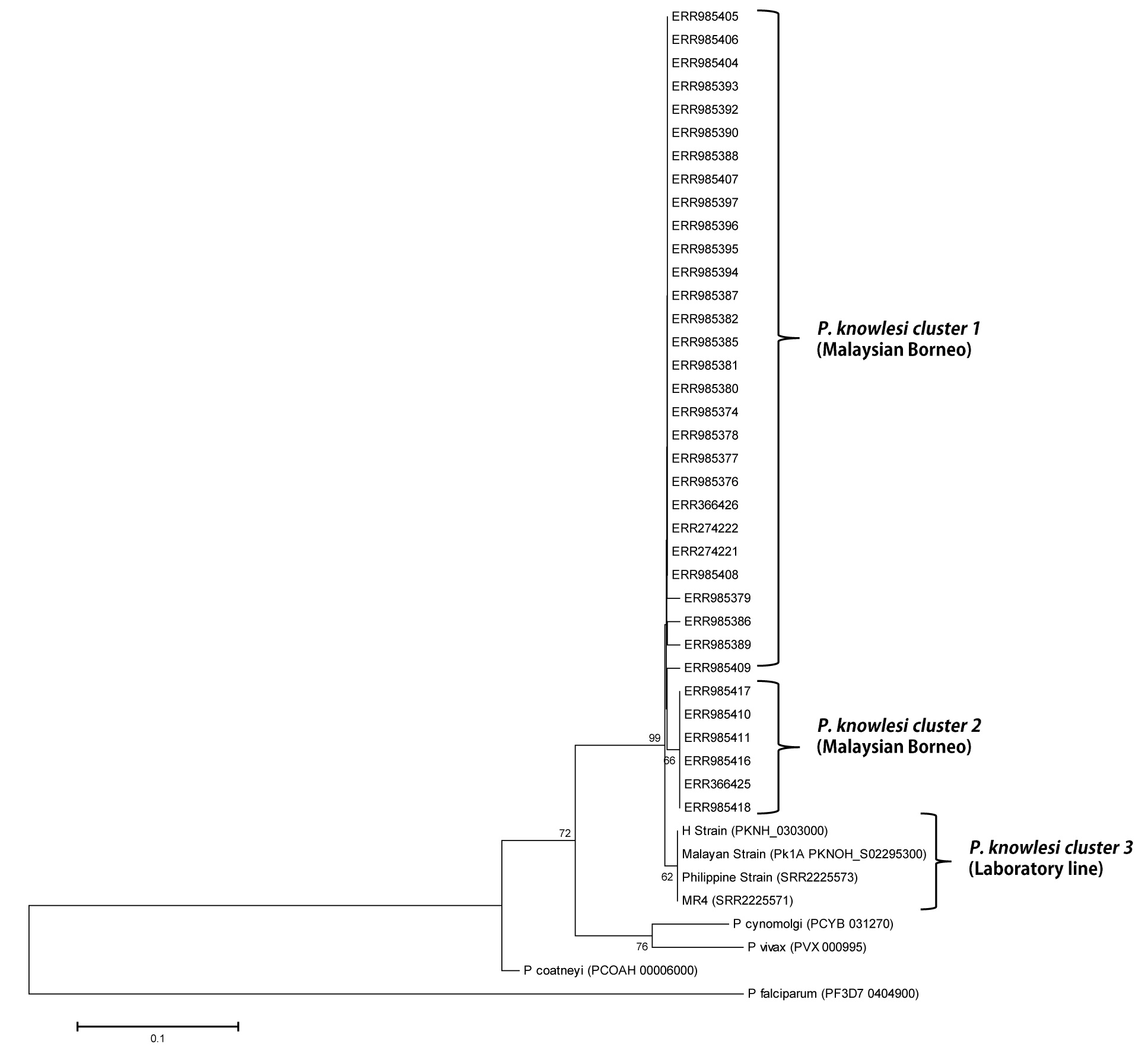

Supplement: Supplemental Information 5 — The two P. knowlesi sub-populations identified based on s48/45 domain I in Malaysian Borneo are shown as cluster 1 and cluster 2 and the four laboratory lines formed the cluster 3 from Peninsular Malaysia. Numbers at the nodes indicate bootstrap values. [file peerj-06-6141-s005.png]

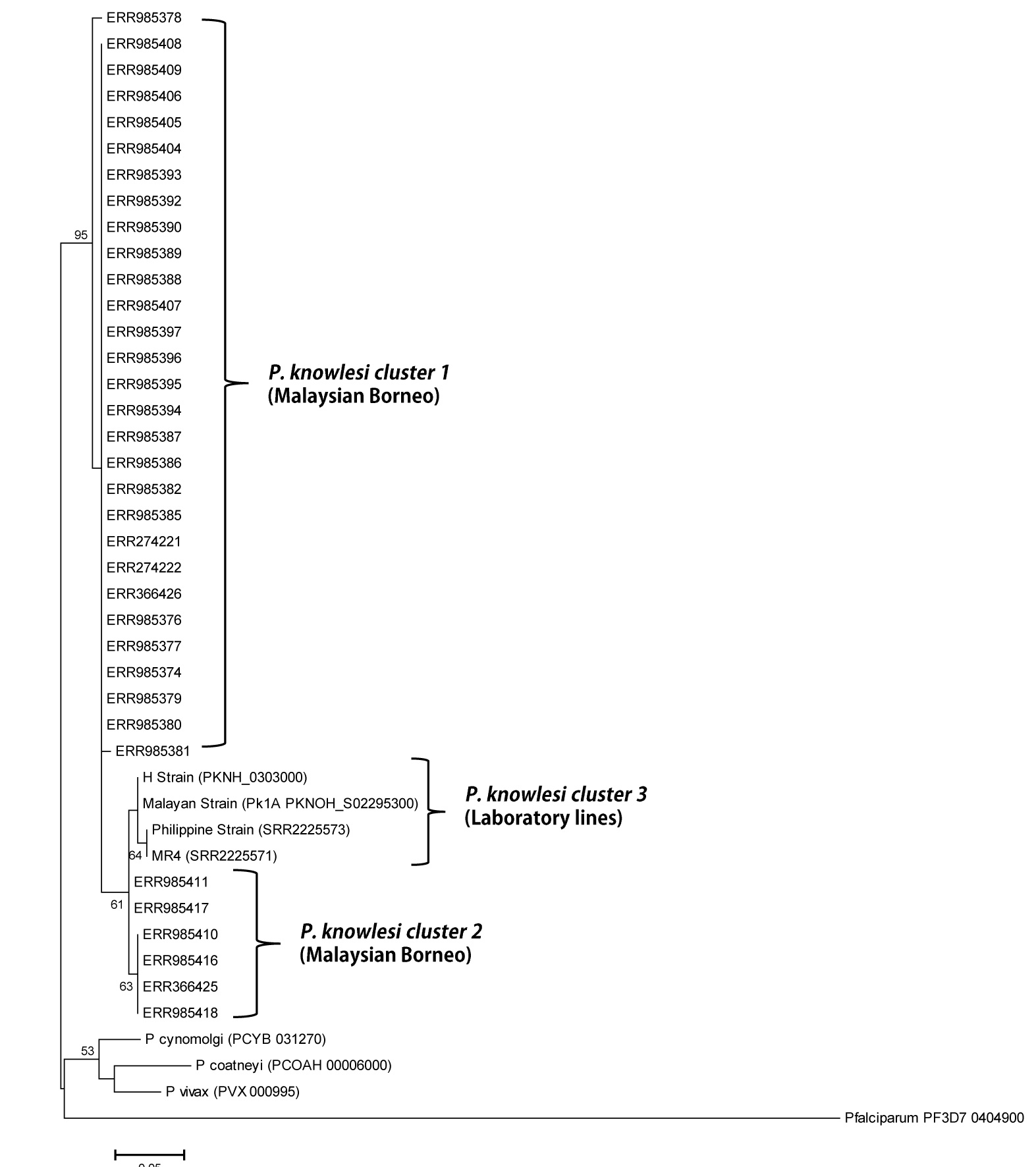

Supplement: Supplemental Information 6 — The two P. knowlesi sub-populations identified based on s48/45 domain II in Malaysian Borneo are shown as cluster 1 and cluster 2 and the four laboratory lines formed the cluster 3 from Peninsular Malaysia. Numbers at the nodes indicate bootstrap values. [file peerj-06-6141-s006.png]
