# Supplementary material for: The Plasmodium knowlesi Pk41 surface protein diversity, natural selection, sub population and geographical clustering: a 6-cysteine protein family member
Source: PeerJ. 2018 Dec 14;6:e6141. doi: 10.7717/peerj.6141 (PMC6296336; doi:10.7717/peerj.6141)
Supplement: Supplemental Information 7 — P: Peninsular. [file peerj-06-6141-s007.docx]

Supplementary Table S1. Study samples and origin

| No. | Sample | Area | Year |
| --- | --- | --- | --- |
| 1 | ERR274221 | Sarikei | 2012/3 |
| 2 | ERR274222 | Sarikei | 2012/3 |
| 3 | ERR366425 | Sarikei | 2012/3 |
| 4 | ERR366426 | Sarikei | 2012/3 |
| 5 | ERR985374 | Betong | 2012/3 |
| 6 | ERR985376 | Betong | 2012/3 |
| 7 | ERR985377 | Betong | 2012/3 |
| 8 | ERR985378 | Betong | 2012/3 |
| 9 | ERR985379 | Betong | 2012/3 |
| 10 | ERR985380 | Betong | 2012/3 |
| 11 | ERR985381 | Betong | 2012/3 |
| 12 | ERR985382 | Betong | 2012/3 |
| 13 | ERR985385 | Kapit | 2012/3 |
| 14 | ERR985386 | Kapit | 2012/3 |
| 15 | ERR985387 | Kapit | 2012/3 |
| 16 | ERR985388 | Kapit | 2012/3 |
| 17 | ERR985390 | Kapit | 2012/3 |
| 18 | ERR985392 | Kapit | 2012/3 |
| 19 | ERR985393 | Kapit | 2012/3 |
| 20 | ERR985394 | Kapit | 2012/3 |
| 21 | ERR985395 | Kapit | 2012/3 |
| 22 | ERR985396 | Kapit | 2012/3 |
| 23 | ERR985397 | Kapit | 2012/3 |
| 24 | ERR985404 | Kapit | 2012/3 |
| 25 | ERR985405 | Kapit | 2012/3 |
| 26 | ERR985406 | Kapit | 2012/3 |
| 27 | ERR985407 | Kapit | 2012/3 |
| 28 | ERR985408 | Kapit | 2012/3 |
| 29 | ERR985409 | Kapit | 2012/3 |
| 30 | ERR985410 | Betong | 2012/3 |
| 31 | ERR985411 | Betong | 2012/3 |
| 32 | ERR985416 | Kapit | 2012/3 |
| 33 | ERR985417 | Kapit | 2012/3 |
| 34 | ERR985418 | Kapit | 2012/3 |
| 35 | SRR2225571 (MR4) | P. Malaysia |  |
| 36 | SRR2225573 (Philippine) | P. Malaysia |  |
| 37 | Malayan Strain Pk1A [PKNOH_S02295300](http://plasmodb.org/plasmo/app/record/gene/PKNOH_S02295300) | P. Malaysia |  |
| 38 | H-strain(PKNH_0303000) | P. Malaysia |  |
| 39 | ERR985389 | Kapit | 2012/3 |

P: Peninsular
