# Supplementary material for: The Plasmodium knowlesi Pk41 surface protein diversity, natural selection, sub population and geographical clustering: a 6-cysteine protein family member
Source: PeerJ. 2018 Dec 14;6:e6141. doi: 10.7717/peerj.6141 (PMC6296336; doi:10.7717/peerj.6141)
Supplement: Supplemental Information 8 [file peerj-06-6141-s008.docx]

**Table S2**

**Haplotype Frequency**

H_1 ATGTTGGCCGAATTGAGTGCAAAGACTTCGCCCGAGACCCCGGTCA 2

H_2 ATGCTGGCCGAATTGAGTGCAAAGACTTCGCCCGAGACCCCGGAAG 2

H_3 ATGCTGGCCGAACCGAGCGTGGAGAAAGAGGATGAGAACCCCGTAG 1

H_4 ATGCTGGCCGAGTCGCACGTGGGGAAAGAGGATGAGACCCCCGTAG 1

H_5 ATGTTGGCCGAATTGAGCACGGGGAAAGGGGCCGAAACCCCGGTCG 2

H_6 ATGTTGGCCGAATTGAGCACGGGGAAAGGGGCCGAAACCCCGATCG 3

H_7 ATGCTGGCCGAACTGAGCGTGGAGAAAGAGGATGAGAACCCCGTAG 3

H_8 ATGCTGGCCGAACTGAGCGTGGAGAAAGAGGATGAGACCCCCGTAG 2

H_9 ATGCTGGCCGAACTGAGCGTGGGGAAAGAGGATAAGACCCCCGTAG 1

H_10 ATGCTGGCCGAACCGAGCGTGGAGAAAGAAGATGAGGACCCCGTAG 1

H_11 ATGCTAGCCGAACTGAGCGTGGAGAAAGAGGATAAGAACCCCGTAG 1

H_12 ATGCTGTCCGGACTAAGCGTGGAGAAAGAGGATGAGAACCCCGTAG 1

H_13 ATGCTGGCCGAACCGCGCGTGGGGAAAGAGGATGAGACCCCCGTAG 1

H_14 ATGCTGGCCGAACTGCACGTGGGGAAAGAGGATAGGACCCCCGTAG 1

H_15 ATGCTGGCCGAACTGAGCGTGGGGAAAGAGGATGAGACCCCCGTAG 1

H_16 ATGCTGGCCGAACTAAGCGTGGAGAAAGAGGATGAGACCCCCGTAG 1

H_17 ATGCTGGCGGAACCGAGCGTGGAGAAAGAGGATGAGAACCCCGTAG 1

H_18 ATGCTGGCCAAACTGAGCGTGGGGAAAGAGGATGAGACCCCCGTAG 1

H_19 ATGCTGGCCGGACCGAGCGTGGGGAAAGAGGATGAGACCCCCGTAG 1

H_20 ATGCTGGCCGAATTGAGCGTGGGGAAAGAGGATGAAATCCCCGTAG 1

H_21 ATGCTGGCCGAACCGAGCGTGGGGAAAGAGGATGAGAACCCCGTAG 1

H_22 ATGCTGGCCGAACTAAGCGTGGGGAAAGAGGATGAGACCCCCGTAG 1

H_23 ATGCTGGTCGAACTGAGCGTGGGGAAAGAGGATAAGACCCCCGTAG 1

H_24 ATGCTGGCCGGATTGAGCGTGGGGAAAGAGGATGAGACCCTCGTAG 1

H_25 ATGCTGGCCGAACTGCACGTGGGGAAAGAGGATGAGAACCCCGTAG 1

H_26 ATGCTGGCCGGACTGAGCGTGGGAAAAGAGGATGAGAACCCCGTAG 1

H_27 ATGCTGGCCGAACCGCGCGTGGGGAAAGAGGATGAGAACCCCGTAG 1

H_28 ATGCTGGCCGAACTGAGCGTGGGGAAAGAGGATGAGACATCCGTAG 1

H_29 ATGCTGGCCGAACTGAGCGTGGGGAAAGAGGATAAGAACCCCGTAG 1

H_30 ATGCCGGCCGAACTGAGCGTGGAGAAAGAGGATAAGAACCCCGTAG 1

H_31 TCTTTGGCCGAATTGAGCACGGGGGAAAAGGCCGAAACCCCGATCG 1
