# Supplementary material for: The Plasmodium knowlesi Pk41 surface protein diversity, natural selection, sub population and geographical clustering: a 6-cysteine protein family member
Source: PeerJ. 2018 Dec 14;6:e6141. doi: 10.7717/peerj.6141 (PMC6296336; doi:10.7717/peerj.6141)
Supplement: Supplemental Information 9 [file peerj-06-6141-s009.docx]

**Table S3: Population differentiation values (*F_ST_*) from each sub-populations of Malaysia based on *Pk41* genes**

| Location | *F_ST_* values* | | |
| --- | --- | --- | --- |
|  | Cluster 1 Malaysian Borneo (Sarawak) | Cluster 2 Malaysian Borneo (Sarawak) | Cluster 3 Peninsular Malaysia |
| Cluster 1 Malaysian Borneo (Sarawak) | - |  |  |
| Cluster 2 Malaysian Borneo (Sarawak) | 0.732** | - |  |
| Cluster 3 Laboratory lines | 0.785** | 0.819** | - |

** *P < 0.000*
